# Assessment of nutritional change processes and their relationship with macronutrient intake and anthropometric measurements in adults

**DOI:** 10.3389/fnut.2026.1728715

**Published:** 2026-02-11

**Authors:** Beyza Nur Seker, Neslihan Kocatepe, Damla Zeynep Bayraktar, Hande Seven Avuk

**Affiliations:** 1 Department of Nutrition and Dietetics, Istanbul Bilgi University Institute of Graduate Programs, Istanbul, Türkiye; 2 Department of Nutrition and Dietetics, Faculty of Health Sciences, Istanbul Bilgi University, Istanbul, Türkiye

**Keywords:** body mass index, body weight changes, diet, transtheoretical model, weight loss

## Abstract

**Background:**

This study assessed the relationship between adults' nutritional behavior change process and their diet implementation, energy, macronutrient intake, and anthropometric measurements.

**Methods:**

This cross-sectional study was conducted with 400 adults (50% men, 50% women, median age 27 years). A face-to-face questionnaire including sociodemographic information, smoking status, diet experience, and the Nutritional Change Processes Scale (NCPS) was applied. Food consumption was recorded using a 24-hour recall method, and anthropometric measurements were taken.

**Results:**

Among participants, 50.5% were classified as underweight-normal, 33.5% as overweight, and 16% as obese, while 37.8% had past dieting experience. Those with a dieting history had significantly higher NCPS scores [110 (52–192)] than those without [77 (48–176); *p* < 0.001]. The NCPS score of obese participants [113.50 (52–192)] was the highest compared to underweight-normal [79.50 (48–177)] and overweight [90.5 (48–176)] participants (*p* < 0.001). A positive correlation was found between total NCPS score and body weight, BMI, and body fat percentage (*r* = 0.200, *p* < 0.001; *r* = 0.355, *p* < 0.001; *r* = 0.161, *p* = 0.001, respectively). A negative correlation was observed with energy intake, carbohydrates, and carbohydrate percentage (*r* = −0.132, *p* = 0.008; *r* = −0.165, *p* = 0.001; *r* = −0.158, *p* = 0.002, respectively). However, in multivariable analyses, the association between BMI and NCPS was no longer significant after adjustment for age and gender, and was significantly influenced by energy intake (*p* < 0.05).

**Conclusion:**

This study shows that obesity and past dieting experiences are associated with higher involvement in dietary behavior change processes, but this relationship is significantly influenced by age, gender, and energy intake. Furthermore, the finding that high awareness does not guarantee balanced macronutrient intake underscores the need for multidimensional weight management strategies that address both individual cognitive factors and environmental determinants.

## Introduction

Chronic diseases, obesity, and cognitive disorders are among the most important health problems faced by both developed and developing countries in recent years. In addition to genetic factors playing a role in developing these diseases, lifestyle modification interventions also significantly reduce these risks (1, 2). It is known that mortality and morbidity rates decrease with behavioral changes, and the quality of life of individuals increases (3). The World Health Organization (WHO) emphasizes the importance of healthy dietary practices in preventing non-communicable diseases (4).

Diet programs are generally based on limiting energy intake, and individuals are encouraged to make changes in their eating habits in order for these programs to be effective throughout life (5). A weight loss program aims to reduce body weight by 5–10%, significantly improving blood pressure, cholesterol, and glucose levels. However, it is also critical to achieve a 5% reduction in body weight and to maintain this in the long term (6).

High rates of dieting can lead to weight cycles and adverse health outcomes in individuals (7). In the general population, individuals with normal weight have higher rates of weight cycling observed (8). Some studies show that individuals with a history of weight cycling have a higher prevalence of obesity (9, 10). Weight cycling is an important risk factor for metabolic and cardiovascular diseases (11). A study examining the effect of weight cycling on cardiometabolic risk factors in women reported that anthropometric risk factors such as BMI, waist and hip circumference, waist/hip ratio, body fat percentage, fat mass, and biochemical cardiometabolic risk factors such as total cholesterol, LDL-cholesterol and triglyceride levels were higher in weight cycling individuals compared to non-weight cycling individuals (12). Individuals with normal body weight, as well as individuals with overweight problems, often apply diet for weight loss (13). In a cross-sectional study, 11% of individuals were found overweight, and 29.7% of women and 8.3% of men were found to diet frequently (14).

Nutrition behavior change is a complex process influenced by nutrition knowledge and psychosocial factors such as intention, perceived barriers, perceived risks, self-efficacy, outcome expectations, and self-control (15). In a study by Prochaska et al. examining 12 different areas of behavior change within the Transtheoretical Model (TTM) framework, the relationships between stages of change and decision balance were focused. Regarding dietary behavior, individuals go through a decision-making process before changing behavior, and the pros and cons of this process shape their perceptions toward the change reported. In the study, the balance of pros and cons differed according to the change stages of individuals stated. This model is important in understanding nutritional behavior change and determining which type of support individuals need at which stage (16). In another study in which Prochaska et al. examined the effects of TTM on health behavior changes, they emphasized that the process of behavior change is gradual, and individuals need different interventions at different stages. It was stated that the ability of individuals to sustain change in nutrition behavior depends on self-efficacy, decision balance, and change processes. The study shows that individuals' intrinsic motivation and environmental support are important in gaining healthy eating habits. It is emphasized that individuals should be guided by appropriate support systems and progressive interventions in order to make the change permanent (17). Furthermore, a review study examining models of dietary behavior change showed that dietary interventions based on the transtheoretical model could effectively reduce dietary fat intake and increase fruit and vegetable consumption (18). This study aimed to investigate the relationship between energy, macronutrient consumption, and anthropometric measurements and the process of changing nutritional behavior toward dietary practice in adults.

## Materials and methods

### Study design

The sample of this cross-sectional study conducted between January and October 2023 consisted of 400 adults aged 18–65 years who were living in Kahramanmaraş province of Türkiye and met the research criteria. Individuals under the age of 18 and over the age of 65, adults residing outside the province of Kahramanmaraş, pregnant and breastfeeding women, individuals with special dietary needs, and individuals who did not fill out the informed consent form were not included in our study. According to the 2021 “Address Based Population Registration System” data of the Turkish Statistical Institute (TurkStat), there are a total of 699,700 individuals, 357,250 males (51%) and 342,450 females (49%), between the ages of 18–65 in Kahramanmaraş province (19). Study participants were selected by simple random sampling within a stratified sampling design to ensure appropriate representation of the target population. Based on initial power calculations, a minimum of 384 individuals (196 males and 188 females) were required. In an effort to maximize the robustness of the statistical analyses and increase the power, the total sample was deliberately expanded, resulting in a final cohort of 400 adults (200 males and 200 females) included in the final data analysis. This study was conducted according to the guidelines laid down in the Declaration of Helsinki and all procedures involving research study participants were approved by the Human Research Ethics Committee of Istanbul Bilgi University (Project No: 2023-20160-009, Date: 17.01.2023). Following the verbal explanation of the study, voluntary consent forms were obtained from the participants who agreed to take part.

### Data collection tools

The survey form was prepared by the researcher and was applied face-to-face. The face-to-face survey and data collection, including all anthropometric measurements, were conducted in a controlled environment at a specialized dietitian‘s office in Kahramanmaraş. This environment ensured the standardization and confidentiality necessary for accurate measurements and the maintenance of detailed 24-hour dietary records. The survey consisted of three sections: the first section included sociodemographic characteristics and dietary habits; the second section included anthropometric measurements and 24-hour food consumption records; and the third section included the Nutritional Change Processes Scale (NCPS).

### Anthropometric measurements

Body weight, height, waist, and hip circumference measurements of the individuals were measured in light clothing on an empty stomach in accordance with anthropometric measurement standards (20). The waist/hip ratio was evaluated according to WHO data (21). The body mass index (BMI) of the individuals was obtained by dividing the square of the height by the body weight (kg/m^2^) (22). Based on BMI values, participants were categorized into three groups according to WHO classifications. For the purpose of discussion and presentation in the results section, these groups are referred to as: underweight and normal (< 24.99 kg/m^2^), overweight (25–29.99 kg/m^2^), and obese (≥30.00 kg/m^2^).

### 24-Hour dietary recall

Participants‘ energy and macronutrient intake were determined using a visual aid food catalog developed by Rakicioglu and Acar Tek (23) using a 24-hour dietary recall method. The 24-hour recall data were collected on a standard weekday to reflect participants' routine dietary habits and to minimize potential variability associated with weekend consumption patterns. Food consumption records were analyzed using the Nutrition Information Systems (BeBiS) full version 8.1 (Pacific Ltd. Sti., Istanbul, Türkiye).

### Nutritional change processes scale (NCPS)

The NCPS has been developed by Prochaska et al. (24) to determine how experiences affect people‘s eating habits. Validity and reliability studies in Turkish were conducted by Menekli and Fadiloglu (25). The scale consists of 48 items and 12 subscales, and each item of the scale is evaluated with a grading score ranging from one (never) to five (very often). The subscales of this scale are consciousness raising, dramatic help/emotional animation, environmental revaluation, self-reevaluation, social liberation, counterconditioning, helping relationships, reinforcement management, self-liberation, stimulus control, peer-to-peer system control, and drug use. The scores were obtained on a scale between 48 and 240. Cronbach's alpha value in the Turkish adaptation was 0.90 (25).

### Statistical analysis

The data obtained were analyzed using IBM SPSS software (version 28.0, Inc., Chicago). Categorical variables were defined as number (*n*) and percentage (%). The conformity of the data to normal distribution was evaluated using the Kolmogorov-Smirnov test. Descriptive statistics were reported as median and minimum-maximum (min–max) values for data that were not normally distributed. The chi-square test was used to compare categorical variables. Kruskal–Wallis test for three groups and the Mann–Whitney *U*-test for two groups were used to compare the median values of the data that did not fit a normal distribution. When a statistically significant difference was detected, *post hoc* analyses were performed using the pairwise comparisons with Bonferroni correction. The relationships between numerical variables were evaluated with the Spearman's rho correlation coefficient. To further examine the predictors of NCPS, the normality of the data distribution was assessed according to the criteria proposed by George and Mallery (26), where skewness and kurtosis values within the range of ± 1 indicated normal distribution. Initially, a Stepwise Multiple Linear Regression was performed to identify predictors of the NCPS total score. As the strongest predictor, BMI was utilized as the ‘Crude Model' in the subsequent five-stage hierarchical multiple linear regression strategy. Gender, age, energy intake, and macronutrients were sequentially added to subsequent models to evaluate the independent effect of BMI on the total NCPS score. The results are presented as standardized beta (β) coefficients, 95% confidence intervals (CI), and *p*-values. Statistical significance was accepted as *p* < 0.05 in all analyses.

## Results

In this study, which was completed with a total of 400 participants, the number of male and female cases was equal, and the median age was 27 years. It was determined that 37.8% of the individuals had diet experience in the past, and 62.9% had dieted one to four times before (Table 1). The median BMI was 24.85 kg/m^2^. It was found that 50.5% of the individuals were underweight-normal body weight, 33.50% were overweight, and 16% were obese (Table 2). The median daily energy intake was 1,370.2 kcal (carbohydrate: 45%, protein: 15%, and fat: 40%) for females and 2,155.2 kcal (carbohydrate: 47%, protein: 16% and fat: 37%) for males (*p* < 0.001; Table 3).

**Table 1:** Baseline characteristics of participants.

**Variables**	***n***	**%**
**Gender**		
Female	200	50
Male	200	50
**Marital status (** ***n*** **, %)**		
Single	228	57
Married	172	43
**Education status (** ***n*** **, %)**		
Elementary school	33	8.3
High school/secondary education	59	14.7
Undergraduate degree	294	73.5
Graduate degree	14	3.5
**Job status (** ***n*** **, %)**		
Housewife	33	8.2
Self-employed	60	15
Civil servant	85	21.2
Laborer	97	24.3
Retired	5	1.3
Student	118	29.5
Unemployed	2	0.5
**Smoking (** ***n*** **, %)**		
Yes	163	40.8
No	237	59.2
**Past diet experience (** ***n*** **, %)**		
Yes	151	37.8
No	249	62.2
**Frequency of dieting (** ***n*** **, %)**		
1–4 times	95	62.9
5–10 times	22	14.6
More than 10	24	15.9
I'm always on a diet	10	6.6

n, number; %, percent.

**Table 2:** Age, anthropometric measurements and BMI classification of participants.

**Parameters**	**Female (*n* = 200)**	**Male (*n* = 200)**	**Total (*n* = 400)**	***p***
Age (years)	26 [18–60]	28.5 [18–64]	27.00 [18–64]	0.789
Body weight (kg)	63 [40–129.4]	82 [50–140]	73.00 [40–140]	**< 0.001** ^*^
Height (cm)	163 [148–178]	178 [157–197]	170.00 [148–197]	**< 0.001** ^*^
BMI (kg/m^2^)	23.45 [15.5–53.2]	25.8 [16.1–41.8]	24.85 [15.50–53.20]	**< 0.001** ^*^
Waist circumference (cm)	80 [59–156]	95 [65–136]	88.00 [59–156]	**< 0.001** ^*^
Hip circumference (cm)	102 [75–156]	104.5 [83–127]	103.00 [75–156]	0.181
Waist/hip	0.78 [0.63–1.74]	0.9 [0.65–1.1]	0.84 [0.6–1.74]	**< 0.001** ^*^
**BMI category**				
< 25.00 kg/m^2^	121 (60.5)	81 (40.5)	202 (50.5)	**0.001** ^**^
25.00–29.99 kg/m^2^	50 (25.0)^a^	84 (42.0)^b^	134 (33.50)	
≥30.00 kg/m^2^	29 (14.50)	35 (17.50)	64 (16.00)	

*p* < 0.05, number (percentage), median [min–max].

^*^Mann–Whitney U-test.

^**^Chi-Square test.

^a, b^There is a difference between groups with the same letter.

BMI, Body mass index; NCPS, Nutritional change processes scale.

*p* < 0.05 statistically significant results shown in bold.

**Table 3:** Daily energy and macronutrient intake data.

**Energy and nutrients**	**Female (*n* = 200)**	**Male (*n* = 200)**	***p***
Energy (kcal)	1,370.2 (322.56–3,224.25)	2,155.2 (846.35–7,645.23)	**< 0.001**
Carbohydrate (g)	147.1 (27.4–374.6)	234.8 (54–1,234.5)	**< 0.001**
Carbohydrate (%)	45 (18–75)	47 (13–72)	**0.030**
Fiber (g)	15.2 (2.4–38.9)	22.7 (4.1–71.2)	**< 0.001**
Protein (g)	49.6 (6.9–114.1)	80.7 (18–292.8)	**< 0.001**
Protein (%)	15 (4–32)	16 (8–44)	**0.145**
Fat (g)	57.95 (12.49–151.71)	85.51 (23.91–233.14)	**< 0.001**
Fat (%)	40 (12–68)	37 (14–69)	**< 0.001**

*p* < 0.05, median (min–max), Mann–Whitney U-test.

*p* < 0.05 statistically significant results shown in bold.

The NCPS and its subscale scores are based on the presence of past dietary experience, BMI category, and gender, as shown in Table 4. Individuals with a past dietary experience had a significantly higher median NCPS score [110 (52–192)] compared to those without a dietary history [77 (48–176); *p* < 0.001]. This trend was consistent across all subscales of the NCPS, where the median score was also higher in those with a dietary history than in those without (*p* < 0.05). The BMI classification of participants with a past diet experience (*n* = 151) was analyzed to assess the outcome of high NCPS engagement: 33.8% were in the underweight/normal BMI category, while a majority (66.2%) were classified as overweight (37.7%) or obese (28.5%; data not shown). The median NCPS total score of obese individuals [113.50 (52–192)] was significantly higher than that of underweight-normal [79.50 (48–177)] and overweight individuals [90.5 (48–176); *p* < 0.001]. The NCPS total scores of overweight individuals were significantly higher than those of underweight-normal individuals (*p* < 0.001). The mean scores of obese individuals were statistically significantly higher in all subscales except the subscales of environmental reevaluation (*p* < 0.001). The mean NCPS total and subscale scores of females were significantly higher than those of males (*p* < 0.001; Table 4).

**Table 4:** Evaluation of the NCPS according to participants' past diet experience, BMI category and gender.

**NCPS subscales**	Past Diet Experience		*p* ^*^	BMI Category			*p* ^**^	Gender		*p* ^*^	**Total (*n* = 400)**
	**Yes**	**No**		** ≤ 24.99 kg/m^2^**	**25.00–29.99 kg/m^2^**	**≥30 kg/m^2^**		**Female**	**Male**		
	**(*n* = 151)**	**(*n* = 249)**		**(*n* = 202)**	**(*n* = 134)**	**(*n* = 64)**		**(*n* = 200)**	**(*n* = 200)**		
Consciousness raising	9 (4–20)	5 (4–16)	**< 0.001**	6.00 (4–19)^a^	7 (4–19)^b^	9.00 (4–20)^c^	**< 0.001**	7 (4–20)	6 (4–19)	**< 0.001**	7.00 (4–20)
Dramatic help/emotional animation	10 (4–20)	7 (4–18)	**< 0.001**	8.00 (4–16)^a^	9 (4–18)^a^	11.00 (4–20)^b^	**< 0.001**	9 (4–20)	8 (4–18)	**< 0.001**	9.00 (4–20)
Environmental reevaluation	8 (4–20)	7 (4–19)	**0.043**	8.00 (4–19)	7 (4–20)	8.00 (4–17)	0.847	8 (4–20)	7 (4–19)	**0.018**	8.00 (4–20)
Self-reevaluation	11 (4–20)	6 (4–19)	**< 0.001**	6.00 (4–17)^a^	8 (4–20)^b^	12.5 (4–20)^c^	**< 0.001**	8 (4–20)	7 (4–19)	**0.009**	8.00 (4–20)
Social liberation	11 (4–20)	8 (4–19)	**< 0.001**	8.00 (4–17)^a^	9 (4–19)^a^	11.00 (4–20)^b^	**< 0.001**	10 (4–20)	9 (4–17)	**0.001**	9.00 (4–20)
Counterconditioning	9 (4–16)	7 (4–15)	**< 0.001**	7.00 (4–15)^a^	8 (4–16)^ab^	9.00 (4–15)^b^	**0.003**	8 (4–15)	7 (4–16)	**0.001**	8.00 (4–16)
Helping relationships	11 (4–20)	7 (4–20)	**< 0.001**	8.00 (4–20)^a^	9 (4–20)^a^	11.00 (4–20)^b^	**< 0.001**	9 (4–20)	8 (4–20)	**0.002**	8.00 (4–20)
Reinforcement management	7 (4–19)	5 (4–15)	**< 0.001**	4.00 (4–17)^a^	6 (4–17)^a^	7.00 (4–19)^b^	**< 0.001**	6 (4–19)	5 (4–15)	**0.026**	5.00 (4–19)
Self-liberation	12 (4–20)	8 (4–20)	**< 0.001**	8.00 (4–20)^a^	10 (4–19)^b^	13.00 (5–20)^c^	**< 0.001**	10 (4–20)	8 (4–20)	**0.001**	9.00 (4–20)
Stimulus control	6 (4–20)	4 (4–14)	**< 0.001**	4.00 (4–17)^a^	5 (4–15)^a^	6.00 (4–20)^b^	**< 0.001**	4 (4–20)	4 (4–15)	0.165	4 (4–20)
Peer-to-peer system control	7 (4–20)	6 (4–15)	**< 0.001**	6.00 (4–14)^a^	6 (4–15)^a^	8.00 (4–20)^b^	**< 0.001**	7 (4–20)	6 (4–14)	**0.028**	6.00 (4–20)
Drug use	4 (4–15)	4 (4–16)	**< 0.001**	4.00 (4–13)^a^	4 (4–16)^a^	4.00 (4–13)^b^	**< 0.001**	4 (4–15)	4 (4–16)	**0.001**	4.00 (4–16)
NCPS score	110 (52–192)	77 (48–176)	**< 0.001**	79.50 (48–177)^a^	90.5 (48–176)^b^	113.50 (52–192)^c^	**< 0.001**	94 (51–192)	82.5 (48–171)	**< 0.001**	88 (48–192)

*p* < 0.05 istatistical signifinance, median (min–max).

p^*^Mann–Whitney U-test.

p^**^Kruskal–Wallis test; *post hoc*: Bonferroni correction.

^a−*c*^The presence of at least one common letter in the same row indicates that there is no statistically significant difference.

BMI, Body mass index.

*p* < 0.05 statistically significant results shown in bold.

While there was a positive correlation between NCPS total mean score and body weight, BMI and fat (%TE; *r* = 0.200, *p* < 0.001; *r* = 0.355, *p* < 0.001, *r* = 0.161, *p* = 0.001, respectively), a negative correlation was found between energy, carbohydrate (g) and carbohydrate (%TE; *r* = −0.132, *p* = 0.008; *r* = −0.165, *p* = 0.001; *r* = −0.158, *p* = 0.002, respectively). Between increasing BMI levels and all subscales of the NCPS except the subscale of environmental reevaluation, a statistically significant positive correlation was found (*p* < 0.001; Table 5).

**Table 5:** Correlation of NCPS with participants' anthropometric measurements, energy and macronutrient intake data.

**NCPS subscales**		**Body Weight (kg)**	**BMI (kg/m^2^)**	**Energy (kcal)**	**CHO (g)**	**CHO (% TE)**	**Protein (g)**	**Protein (% TE)**	**Fat (g)**	**Fat (%TE)**	**Fiber (g)**
Consciousness raising	*r*	0.173	0.300	−0.178	−0.196	−0.130	−0.132	0.041	−0.100	0.140	−0.050
	*p*	**0.001**	**< 0.001**	**< 0.001**	**< 0.001**	**0.009**	**0.008**	0.409	**0.045**	**0.005**	0.318
Dramatic help/emotional animation	*r*	0.126	0.267	−0.140	−0.174	−0.152	−0.106	0.020	−0.050	0.173	−0.030
	*p*	**0.012**	**< 0.001**	**0.005**	**< 0.001**	**0.002**	**0.034**	0.687	0.318	**< 0.001**	0.551
Environmental reevaluation	*r*	−0.072	−0.007	−0.070	−0.054	−0.006	−0.065	−0.014	−0.064	0.016	0.023
	*p*	0.151	0.886	0.163	0.284	0.899	0.197	0.786	0.201	0.749	0.646
Self-reevaluation	*r*	0.356	0.497	−0.103	−0.150	−0.183	−0.036	0.126	−0.012	0.172	−0.043
	*p*	**< 0.001**	**< 0.001**	**0.040**	**0.003**	**< 0.001**	0.470	**0.012**	0.818	**0.001**	0.392
Social liberation	*r*	0.112	0.237	−0.087	−0.101	−0.077	−0.057	0.036	−0.034	0.083	−0.034
	*p*	**0.025**	**< 0.001**	0.084	**0.043**	0.125	0.258	0.467	0.493	0.097	0.496
Counterconditioning	*r*	0.047	0.178	−0.013	−0.024	−0.073	0.005	0.039	0.020	0.078	0.037
	*p*	0.349	**< 0.001**	0.795	0.634	0.143	0.926	0.437	0.694	0.119	0.464
Helping relationships	*r*	0.128	0.252	−0.126	−0.120	−0.091	−0.074	0.054	−0.097	0.101	−0.012
	*p*	**0.011**	**< 0.001**	**0.012**	**0.017**	0.069	0.142	0.284	0.052	**0.043**	0.807
Reinforcement management	*r*	0.210	0.317	−0.084	−0.124	−0.157	−0.045	0.062	0.003	0.163	−0.047
	*p*	**< 0.001**	**< 0.001**	0.092	**0.013**	**0.002**	0.374	0.213	0.958	**0.001**	0.353
Self-liberation	*r*	0.277	0.421	−0.132	−0.182	−0.186	−0.056	0.143	−0.046	0.165	−0.050
	*p*	**< 0.001**	**< 0.001**	**0.008**	**< 0.001**	**< 0.001**	0.264	**0.004**	0.356	**0.001**	0.322
Stimulus control	*r*	0.145	0.212	−0.045	−0.045	−0.052	0.001	0.060	−0.029	0.034	0.021
	*p*	**0.004**	**< 0.001**	0.365	0.369	0.301	0.987	0.230	0.563	0.496	0.678
Peer-to-peer system control	*r*	0.125	0.228	−0.085	−0.139	−0.175	−0.059	0.051	−0.001	0.194	−0.038
	*p*	**0.012**	**< 0.001**	0.088	**0.005**	**< 0.001**	0.241	0.309	0.987	**< 0.001**	0.444
Drug use	*r*	0.111	0.212	−0.200	−0.226	−0.122	−0.132	0.080	−0.129	0.116	−0.063
	*p*	**0.026**	**< 0.001**	**< 0.001**	**< 0.001**	**0.015**	**0.008**	0.111	**0.010**	**0.020**	0.207
NCPS	*r*	0.200	0.355	−0.132	−0.165	−0.158	−0.075	0.086	−0.051	0.161	−0.028
	*p*	**< 0.001**	**< 0.001**	**0.008**	**0.001**	**0.002**	0.136	0.087	0.306	**0.001**	0.580

*p* < 0.05, r: spearman's rho correlation coefficient.

BMI, body mass index; TE, Total Energy; CHO, carbohydrate; NCPS, nutritional change process scale.

*p* < 0.05 statistically significant results shown in bold.

The relationship between BMI and NCPS total score is shown in Table 6. In the raw model, a positive and statistically significant relationship was found between BMI and NCPS score (β = 0.381; *p* < 0.001). However, when adjusted for gender and age, this relationship lost its significance (Model 1, *p* > 0.05). In Model 2, where energy intake was also added to the model, a negative and statistically significant relationship was observed between BMI and NCPS score (β = −0.120; *p* = 0.018). After further adjustments for protein, carbohydrate, and fat percentages (Models 3–5), the relationship was not found to be statistically significant (*p* > 0.05).

**Table 6:** Multivariate linear regression models for the effect of BMI on NCPS total score.

**Models**		NCPS total score				
		β	*T*	95% Confidence interval		*p* value
				Lower	Upper	
**BMI**	Crude	0.381	8.226	1.780	2.898	**< 0.001**
	Model 1	−0.085	−1.729	−0.553	0.036	0.085
	Model 2	−0.120	−2.384	−0.008	−0.001	**0.018**
	Model 3	0.014	0.319	−0.503	0.698	0.750
	Model 4	−0.071	−1.418	−0.513	0.083	0.157
	Model 5	0.249	1.662	−0.158	1.883	0.097

Crude model: R2: 0.145; < 0.001.

Model 1: Adjusted for gender, age (R2:0.219; < 0.001).

Model 2: Adjusted for gender, age, energy (R2:0.228; < 0.001).

Model 3: Adjusted for gender, age, energy, Protein (% of TE) (R2:0.226; < 0.001).

Model 4: Adjusted for gender, age, energy, Protein (% of TE), Carbohydrate (% of TE) (R2:0.228; < 0.001).

Model 5: Adjusted for gender, age, energy, Protein (% of TE), Carbohydrate (% of TE), Fat (% of TE) (R2:0.232; < 0.001).

NCPS, Nutritional change processes scale; BMI, Body mass index; TE, Total energy.

*p* < 0.05 statistically significant results shown in bold.

## Discussion

Dietary behaviors are influenced by multidimensional variables such as individual motivation, cognitive processes, environmental factors, and psychosocial factors. These variables play an important role in changing individuals' dietary habits toward dietary practices (27). Nutrition behavior change models can impact nutritional status and body composition by determining individuals' compliance with dietary practices (28). In this context, the present study aims to evaluate the relationship between the process of dietary behavior change toward dietary practice and nutritional status and anthropometric measurements in adult individuals.

The subscales of the NCPS comprehensively assess individuals' nutritional behaviors in terms of awareness, emotional reactions, social and environmental factors, individual motivation, habit change, and support mechanisms (24). In this study, it was found that the NCPS total scores and mean scores in the subscales of the NCPS were higher in individuals who had been on a diet than in individuals who had not been on a diet. These results may be explained by the fact that individuals with diet experience are more conscious, experienced, and more prone to change in terms of healthy eating compared to those who have not dieted. It also suggests that the dieting process strengthens individuals' cognitive and emotional mechanisms to change their eating behaviors. In a study examining the relationship between perceived stress, emotional eating, and eating habits in healthcare workers during the COVID-19 pandemic, the mean NCPS score (109.95 ± 29.79) was significantly higher in the group that changed their diet and eating habits than in the group that did not change their diet and eating habits (95.37 ± 32.05) (29).

Mindful eating is an approach that supports conscious food choices and healthy food change (30). In recent years, studies on the effects of mindful eating on individuals' nutritional status and anthropometric measurements have increased. Karaca and Rakicioglu (31) found that mindful eating and nutritional knowledge levels were associated with anthropometric measurements such as body weight and waist circumference. The present study found that the mean NCPS and all sub-subscale scores of obese individuals were higher than those of underweight-normal individuals. This finding suggests that obese individuals have higher nutritional awareness and cognitive and emotional responses to change processes compared to underweight-normal individuals. Similarly, Laz et al. (32) reported that obese individuals have higher nutritional knowledge and are likelier to participate in healthy weight loss behavior. Obese individuals may make more efforts to adopt healthy eating behaviors, make more conscious efforts to change their eating habits and evaluate the effects of social and environmental factors more deeply in this process. This may be because obese individuals have more experience in body weight control and make more attempts to change their diet. Similar to our study, a cross-sectional and descriptive study conducted on university students showed that the NCPS total score of obese students was significantly higher than that of students with normal body weight (33). The available data in the literature show that obese individuals are more exposed to environments that may lead to obesity risk factors compared to normal-weight individuals (34). In support of the literature, the findings of our study reveal that NCPS scores, which evaluate the effect of dietary behaviors related to social and individual factors, are higher in obese individuals. However, it is essential to interpret this relationship with caution. Multivariate regression analysis (Table 6) clarified that the initial positive correlation between BMI and NCPS was largely driven by demographic confounders, as statistical significance disappeared after adjusting for age and gender. Crucially, when controlling for total energy intake, this relationship became significantly negative. This indicates a ‘suppression effect', where high caloric consumption masks the underlying inverse link between body mass and change processes. Consequently, effective weight management requires addressing the complex interplay of demographics and energy intake, rather than relying solely on increasing cognitive awareness.

Scientific literature consistently highlights gender differences in attitudes and behaviors toward weight management. Research indicates that compared to men, women generally possess more accurate body image perceptions but also experience higher body dissatisfaction, leading to a stronger inclination to attempt weight loss (35). Previous studies on university students (36), recreational fitness participants (37), and white-collar workers (38) have similarly reported higher NCPS scores among women. These findings align with our results, confirming that women are more actively engaged in dietary change processes. Recent studies suggest that this disparity is driven by women's higher susceptibility to emotional factors, such as stress and anxiety, and sociocultural expectations regarding body image, which in turn fuels their greater engagement in nutritional behavior change processes (33, 36, 39, 40).

The process of dietary change in obese individuals is not only limited to the control of calorie intake but is also closely related to psychosocial and behavioral factors (41). In this study, a positive correlation was found between the NCPS total score and subscale scores, such as consciousness-raising, dramatic help/emotional animation, self-reevaluation, social liberation, helping relationships, reinforcement management, self-liberation, and drug use, as well as body weight and BMI. On the other hand, a negative correlation was observed between the NPCS total score and energy and carbohydrate intake levels. Öksüzoglu et al. (38) found that daily energy and carbohydrate intake was lower in individuals with high NCPS scores. The present study's finding of higher scores on dietary change processes in obese individuals also highlights the need for specific interventions that address dietary habits and promote healthier eating behaviors. Conversely, the negative correlations with energy and carbohydrate intake suggest that individuals who change dietary may be more selective in their food choices. Furthermore, they are important indicators that obese individuals are in the process of dietary change in many aspects of their lives to reduce body weight.

In our study, despite the NCPS total score showing an inverse relationship with total energy and carbohydrate intake, a positive correlation was observed between several NCPS subscales and the percentage of energy derived from fat. This finding, which seems contradictory at first glance, suggests that high cognitive awareness may not always be accompanied by optimal dietary balance. Individuals scoring high on subscales like consciousness raising and self-reevaluation demonstrate a strong cognitive and emotional commitment to dieting. However, this motivation might be associated with a dietary pattern where carbohydrate restriction coincides with higher proportional fat intake, rather than a balanced reduction in all macronutrients. This positive association with fat intake highlights a potential disconnect between the intention to change behavior and the overall nutritional quality of the diet. Furthermore, the lack of a significant difference in the environmental reevaluation subscale scores among obese individuals suggests a critical challenge regarding ‘obesogenic environments', which are major determinants of unhealthy dietary behaviors (42). When high awareness is not accompanied by effective environmental restructuring (e.g., removing accessible high-fat foods), individuals may find it difficult to limit fat intake, a pattern that aligns with the observed positive correlation.

It is crucial to acknowledge that the challenge in environmental reassessment observed in our study cannot be considered in isolation from broader structural and socioeconomic dynamics. Individual efforts to change dietary habits are often hampered by ‘obesogenic environments' characterized by the prevalence, affordability, and aggressive marketing of energy-dense, low-nutrient foods (43, 44). Conversely, access to fresh, nutrient-rich foods may be restricted by socioeconomic constraints, making healthy dietary choices a luxury (45). Furthermore, cultural determinants significantly shape eating behaviors; specifically in the Turkish context, socialization and hospitality are so deeply intertwined with food sharing that they often create a conflict between participating in cultural rituals and adhering to dietary restrictions (46). Consequently, sustainable dietary change extends beyond individual cognitive processes; it necessitates policy-level and structural interventions to improve the physical food environment and render healthy dietary choices more accessible and culturally adaptable.

While the current study provides valuable insights into the processes of dietary behavior change and their relationship to nutritional status and anthropometric measurements, it has several limitations. First, the study was conducted in a specific population group, which may limit the generalizability of the findings to larger populations. Different cultural, socioeconomic, and environmental factors may influence dietary behavior and require further research in diverse groups. Second, dietary intake was assessed using a single 24-hour recall method. Although this method was administered by a professional dietitian using visual aids to minimize recall bias, it may not fully reflect the participants' habitual, long-term nutritional intake due to day-to-day variations. Third, while the NCPS comprehensively assessed various factors influencing dietary behavior change, other variables such as genetic predispositions, additional psychosocial influences, or specific health conditions were not assessed and may play an important role in individuals' dietary habits and experiences. Future research should address these limitations using longitudinal designs, diversifying sample populations, and including a broader range of influencing factors to improve understanding of dietary behavior change processes.

In conclusion, this study observed that individuals with a diet history and higher body weight developed more attitudes toward the dietary change processes. However, regression analysis highlights that this relationship is mediated by age, gender, and energy intake, emphasizing the need for personalized interventions. In addition, we determined that having more attitudes toward the dietary change process was inversely related to dietary intake of macronutrients such as energy and carbohydrates. Interventions to change dietary behaviors should focus on increasing individuals' intrinsic motivation and developing sustainable habits. The sustainability of behavioral changes is critical in enabling individuals to restructure their eating habits and achieve long-term health goals. Factors such as reinforcement management, self-liberation, and a supportive social environment help individuals maintain healthy weight management by increasing motivation. In this context, taking a multidisciplinary approach to the process of dietary change can facilitate individuals' transition to a healthier lifestyle by supporting both physiological and psychosocial wellbeing. Therefore, increasing individual awareness and providing supportive interventions are important strategies in obesity management.

## Data Availability

The raw data supporting the conclusions of this article will be made available by the authors, without undue reservation.
